# The complete chloroplast genome of *Lilium paradoxum* Stearn (Liliaceae) from southwestern China

**DOI:** 10.1080/23802359.2024.2432367

**Published:** 2024-11-23

**Authors:** Li Luan, Yu-Mei Yuan, Yue-Liang Jiang, Yu-Xi Wang, Jian-Hua Ma, Qiao-Ling He, Yun-Dong Gao

**Affiliations:** a PowerChina Chengdu Engineering Corporation Limited, Chengdu, Sichuan, China; b CAS Key Laboratory of Mountain Ecological Restoration and Bioresource Utilization & Ecological Restoration and Biodiversity Conservation Key Laboratory of Sichuan Province, Chengdu Institute of Biology, Chinese Academy of Sciences, Chengdu, Sichuan, China

**Keywords:** Liliaceae, *Lilium paradoxum*, chloroplast genome, phylogeny

## Abstract

*Lilium paradoxum*, a herb from southeastern Xizang, China, has its first complete chloroplast genome sequenced using next-generation sequencing. The genome is 151,814 bp, consisting of inverted repeats (IRs; 26,323 bp), small single-copy (SSC; 17,524 bp), and large single-copy regions (LSC; 81,644 bp). It encodes 112 unique genes: 78 protein-coding, 30 tRNA, and 4 rRNA genes. Phylogenetic analysis of 22 genomes shows *L. paradoxum* is closely related to *L. gongshanense, L. apertum,* and *L. souliei*. These findings enhance understanding of *Lilium*'s phylogenetic relationships and evolution, particularly the Nomocharis clade in the Hengduan Mountains.

## Introduction

**Figure 1.**
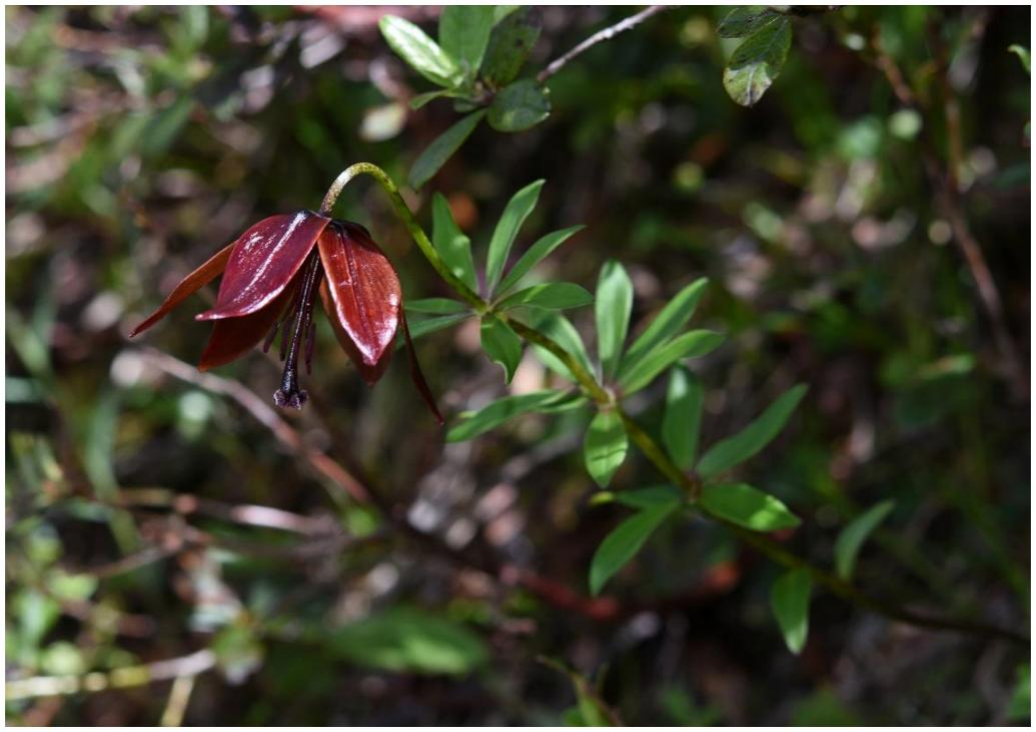
Field picture of *L. paradoxum*, the photo taken by Yun-Dong Gao. *L. paradoxum* grows in shrubbery, featuring purple bell-shaped flowers and delicately symmetrical whorled leaves that are ovate in shape.

*Lilium paradoxum* Stearn (1956), is an herbaceous plant belonging to the genus *Lilium*. It thrives in alpine meadows at elevations surpassing 3200 meters in the southeastern Xizang, with purplish-red, bell-shaped flowers nod gracefully, bearing a striking resemblance to those of *L. souliei* (Franch.) Sealy, distinguished primarily by the exquisite symmetry of its whorled leaves (Figure 1; Stearn 1956; Liang 1980; Liang and Tamura 2000). The genetic relationship based on internal transcribed spacer (ITS) and chloroplast genome fragments reveals that it belongs to Nomocharis clade, which exhibits the contradiction of high divergence in morphology but with low genetic distancing (Liang and Tamura 2000; Gao et al. 2012, 2013, 2015). More genetic information is needed to resolve their phylogenetic relationships. Chloroplasts, vital for photosynthesis, carry genetic information independent of nuclear DNA and encode essential proteins for photosynthesis and biosynthesis (Daniell et al. 2016). With low recombination and nucleotide substitution rates, and typically uniparental inheritance, chloroplast genomes are powerful tools for plant phylogeny studies (Shaw et al. 2005; Tonti-Filippini et al. 2017; Feng et al. 2022).

In the present study, we investigate how *L. paradoxum* contributes to understanding the genetic diversity within the genus *Lilium* by providing an effective phylogenetic framework based on newly assembled and existing chloroplast genome data. We aim to provide critical insights into the evolutionary mechanisms and the diversification within the genus.

## Materials and methods

The fresh leaf samples of *Lilium paradoxum* (Figure 1) were collected from Xizang Province, China (Bomi County: 29.861603° N, 95.766696°E) and dried in silica gel. Voucher specimens were deposited in the Herbarium of the Chengdu Institute of Biology (CDBI) under voucher specimen number *GYD1507* (contact person: Yun-Dong Gao, gaoyd@cib.ac.cn).

DNA was extracted from silica-gel dried leaves using the Doyle and Doyle (1987) protocol. Paired-end sequencing libraries were meticulously constructed with insert sizes of approximately 350 bp, and the sequencing was performed using the DNBseq-4000 platform at the Beijing Genomics Institution (BGI; Shenzhen, China).

About 13 Gb of raw data were filtered by fastp v0.23.2 (Chen et al. 2018) with default parameters and assembled using GetOrganelle v1.7.6.1 (Jin et al. 2020). The chloroplast genome was annotated, and manual corrections were made using Geneious Prime v2023.1.2 (Biomatters Ltd., Auckland, New Zealand) (https://www.geneious.com) and Geseq v.2.03 (Tillich et al. 2017) (https://chlorobox.mpimp-golm.mpg.de/ geseq.html) based on the chloroplast (cp) genome of *L. gongshanense* (NC_052787) and *L. pardanthinum* (MG704135). The chloroplast genome map was generated using CPGview (Liu et al. 2023) (http://www.1kmpg.cn/cpgview).

## Phylogenetic analysis

To clarify the genetic relationship of *Lilium paradoxum*, we reconstructed a phylogenetic analysis based on the whole chloroplast genomes of lily species. We selected 18 complete chloroplasts from various clades of the genus *Lilium*, including the focal species (*L. paradoxum*), 3–5 representative species each from clades Lophophorum and Nomocharis, and 1–2 species from the rest clades. Additionally, we included 3 outgroups from closely related genera. The selection of sequences is based on our previous work (Gao et al. 2012, 2013, 2015).

The sequences were aligned using MAFFT v7.508 (Katoh and Standley 2019). Ambiguously aligned and low mutation sites were filtered using Gblocks v0.9b (Talavera and Castresana 2007) with default parameters. The best nucleotide substitution model was identified using ModelTest-NG v0.1.7 (Darriba et al. 2020). Bayesian Inference (BI) analyses were performed using MrBayes v3.2 (Ronquist et al. 2012) with the GTR+G + I (lset nst = 6 rates = invgamma) model. Posterior probabilities were estimated using two independent Markov Chain Monte Carlo (MCMC) chains (10 million generations), with the initial 25% of sampled data discarded as burn-in. The phylogenetic tree was enhanced using iTOL v6 (https://itol.embl.de).

## Result

**Figure 2.**
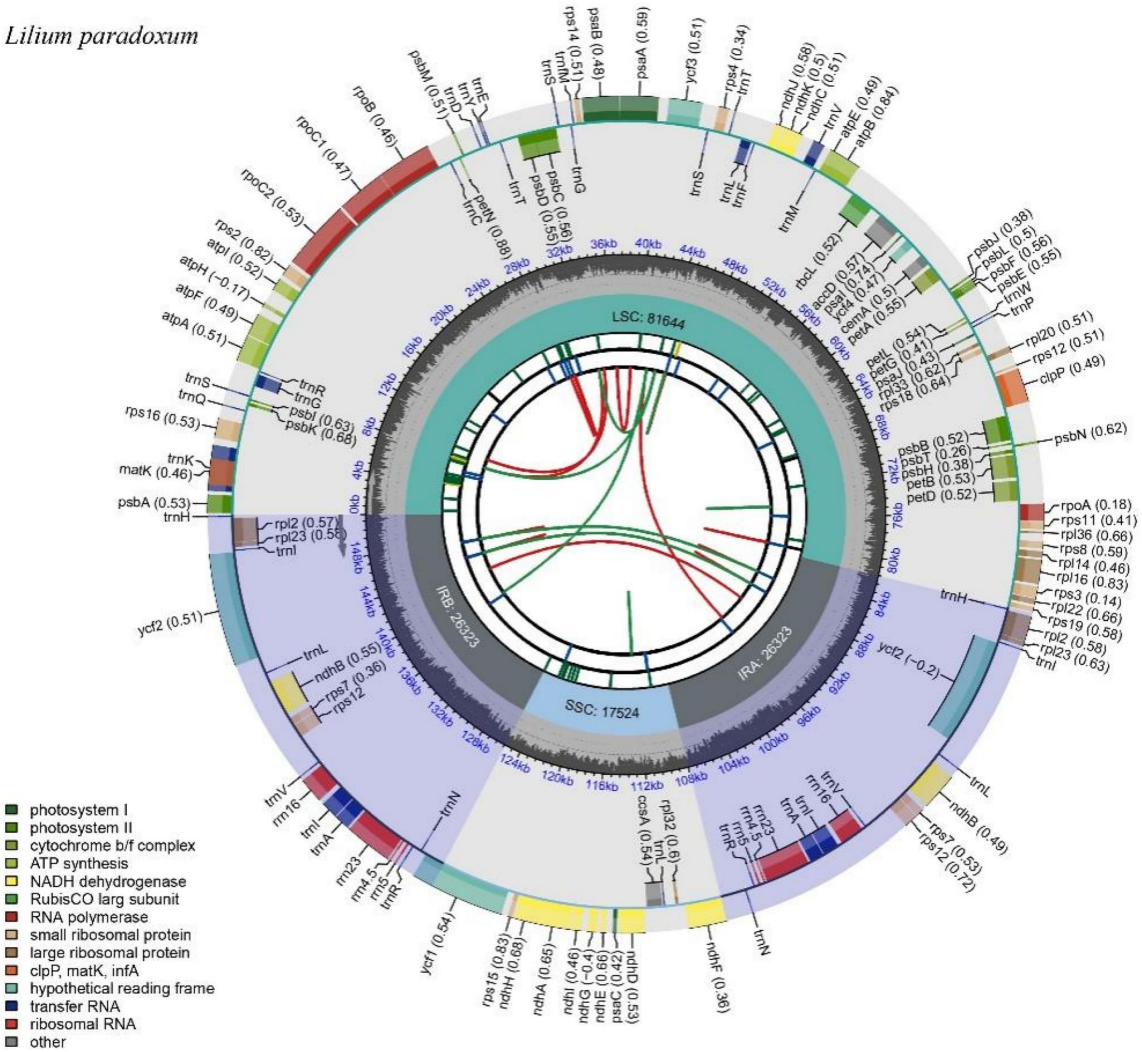
Chloroplast genome map of *L. paradoxum*. The map was generated by CPGView. Genes located on the inner and outer of the circle are transcribed clockwise and anticlockwise, respectively. The dark grey inner circle indicates GC content. Large single-copy (LSC), small single-copy (SSC), and inverted repeats (IRA and IRB) are indicated in the inner layer. The functional classification of the genes is provided in the bottom left corner.

From 12.98 GB of raw data, 12.95 GB was retained after filtering. The raw data are available in the China National Center for Bioinformation (CNCB, https://www.cncb.ac.cn/services) under the BioProject PRJCA022586. The average coverage for the assembled cp genome was 4258× (Figure S1). The complete chloroplast genome of *Lilium paradoxum* was submitted to GenBank under accession number PP073960 and exhibits a typical circular quadripartite structure, composed of a large single-copy (LSC) region of 81,644 bp, a small single-copy (SSC) region of 17,524 bp, and two inverse repeats (IR) regions of 26,323 bp each. The overall GC content stands at 37.0% (Figure 2). A total of 130 genes were annotated (112 unique), including 8 rRNA genes (4 unique), 38 tRNA genes (30 unique), and 84 protein-coding genes (78 unique). Among these genes, the *rps12* gene stands out as a notable trans-splicing gene, characterized by its 5′-exon residing in the LSC region and the other end situated in the IR regions. Additionally, 8 tRNA genes (*trnA-UGC, trnH-GUG, trnI-CAU, trnI-GAU, trnL-CAA, trnR-ACG, trnV-GAC* and *trnN-GUU*), 6 protein-coding genes (*ycf2, ndhB, rpl2, rpl23, rps7* and *rps12*), and 4 rRNA genes (*rrn23S, rrn16, rrn5S* and *rrn4.5S*) were duplicated due to their location within the IR regions (Figure S2 and S3).

**Figure 3.**
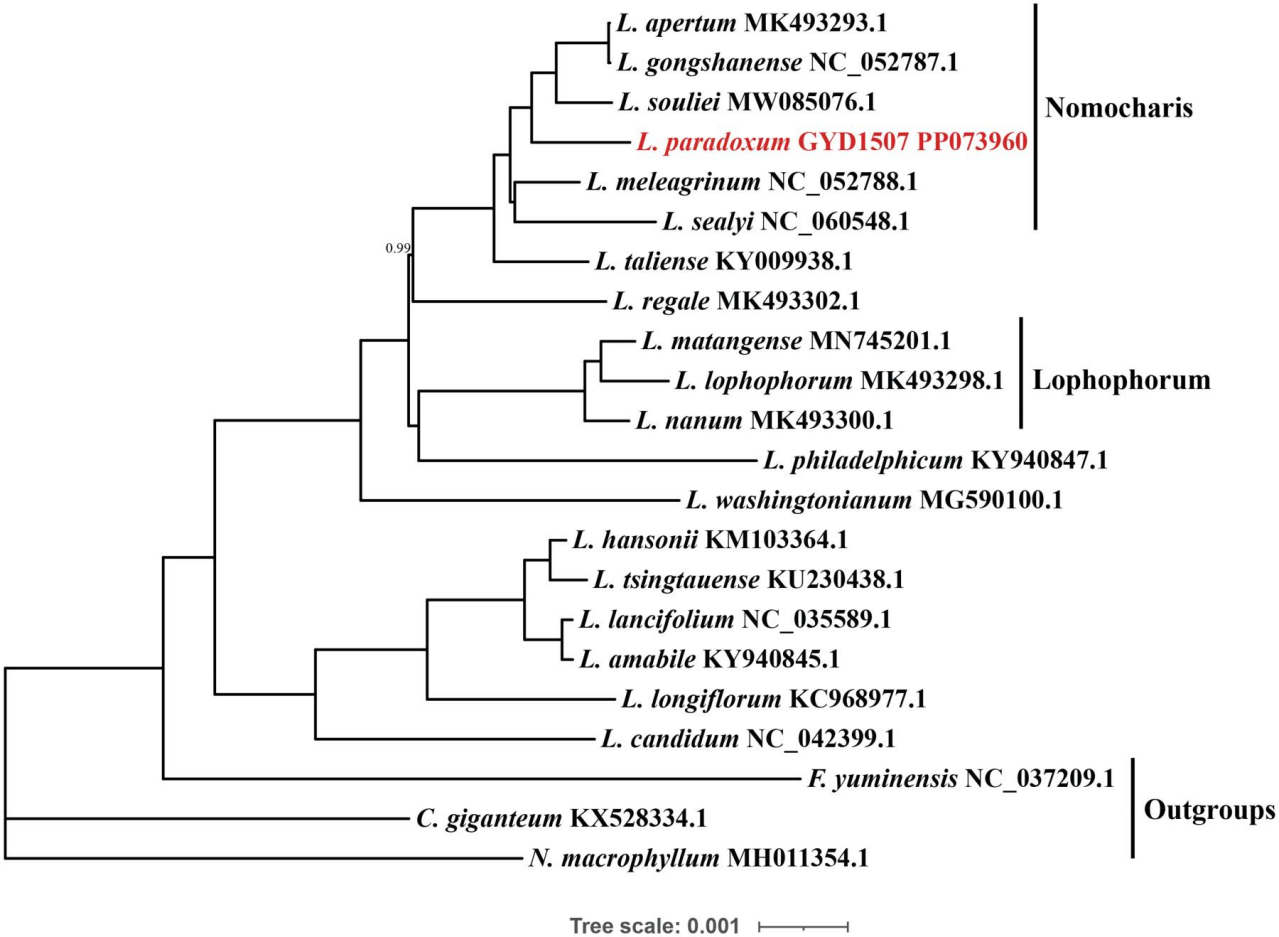
The phylogenetic relationships in 22 representative species in *Lilium* using Bayesian Inference (BI) based on whole plastome sequence data. Bayesian posterior probabilities (PP) on a branch, and PP =1.00 not displayed. *L. paradoxum* are marked in red. Sequences used for tree construction were as follows: *L. amabile,* KY940845.1 (Kim et al. 2017); *L. apertum*, MK493293.1; *L. candidum*, NC_042399.1 (Kim et al. 2019); *L. gongshanense*, NC_052787.1; *L. hansonii*, KM103364.1 (Kim et al. 2016); *L. lancifolium*, NC_035589.1 (Du et al. 2017); *L. longiflorum*, KC968977.1 (Du et al. 2017); *L. lophophorum*, MK493298.1 (Su et al. 2021); *L. matangense*, MN745201.1 (Su et al. 2021); *L. meleagrinum*, NC_052788.1; *L. nanum*, MK493300.1 (Su et al. 2021); *L. paradoxum*, GYD1507 PP073960; *L. philadelphicum*, KY940847.1 (Kim et al. 2017); *L. regale*, MK493302.1; *L. sealyi*, NC_060548.1 (Li et al. 2021); *L. souliei*, MW085076.1; *L. taliense*, KY009938.1 (Zhang et al. 2017); *L. tsingtauense*, KU230438.1 (Song et al. 2016); *L. washingtonianum*, MG590100.1 (Kim and Lim 2018); Outgroups: *Fritillaria yuminensis*, NC_037209.1 (Li et al. 2017); *Cardiocrinum giganteum*, KX528334.1 (Lu et al. 2016); *Notholition macrophyllum*, MH011354.1 (Li et al. 2018).

To clarify the evolutionary and position of *L. paradoxum*, we utilized three closely related genus species as outgroups and constructed Bayesian Inference (BI) tree based on 18 representative lilies from different clades in the genus *Lilium*, and the results (Figure 3) mostly consistent with previous studies (Gao et al. 2012, 2013, 2015). The clade Nomocharis and Lophophorum form two well-supported monophyly, and *L. paradoxum* is in the former, being most closely related to *L. gongshanense* (Y.D. Gao et X.J. He) Y.D. Gao*, L. apertum* Franch. 1898 and *L. souliei*. *L. paradoxum* and its closest relatives form a distinct clade, with a well-resolved phylogenetic relationship (PP = 1.00).

These findings affirm the distinct placement of *L. paradoxum* within the Nomocharis clade and its close phylogenetic relationship with *L. gongshanense*, *L. apertum*, and *L. souliei*, corroborating previous phylogenetic research.

## Discussion and conclusions

The detailed chloroplast genome analysis reveals a typical circular quadripartite structure, with notable gene duplication within the inverse repeat regions. The duplication includes 8 tRNA genes, 6 protein-coding genes, and 4 rRNA genes, which may contribute to the robustness and adaptability of *L. paradoxum*. The concordance between the chloroplast genome data and the phylogenetic tree underscores the evolutionary significance of *L. paradoxum*’s placement and enhances our understanding of the genetic underpinnings behind its unique morphological and ecological adaptations (Liao et al. 2021; Feng et al. 2022; Yuan and Gao 2024). The genomic data not only support the placement of *L. paradoxum* within the Nomocharis clade but also shed light on its close phylogenetic relationships with *L. gongshanense*, *L. apertum*, and *L. souliei* (Figure 3). While the IR regions are more conserved, the LSC and SSC regions show higher nucleotide polymorphism among these species (Yuan and Gao 2024).

This study also provides a genetic basis for parallel evolution within the genus *Lilium*, particularly in the Nomocharis clade (Sun et al. 2012; Zhang et al. 2014; Gao et al. 2015; Yuan and Gao 2024). Closely related species exhibit similar traits due to environmental pressures. For instance, lily species located at higher altitudes, where they are exposed to heavy rain and strong winds, tend to have bell-shaped flowers that protect their fragile reproductive structures, while species found in shrublands at lower elevations often favor saucer-shaped flowers to attract pollinators (Gao et al. 2015; Lawson and Rands 2019; Yuan and Gao 2024).

Overall, this first reported cp genome of *L. paradoxum* is a significant addition to the plastid genome for the whole genus *Lilium*, elucidates the evolutionary position of *L. paradoxum* and lays the groundwork for subsequent studies on the phylogeny, as well as the unique Nomocharis clade species endemic to the Hengduan mountains.

## Supplementary Material

Supplemental Material

## Data Availability

The data that support the findings of this study are openly available in GenBank number PP073960 (https://www.ncbi.nlm.nih.gov/nuccore/PP073960) and the related BioProject, raw sequencing files in SRA, and the Bio-Sample number are PRJCA022586, CRR1005869 and SAMC3300506 (https://www.cncb.ac.cn/services), respectively.
