# Supplementary material for: The complete chloroplast genome of Lilium paradoxum Stearn (Liliaceae) from southwestern China
Source: Mitochondrial DNA B Resour. 2024 Nov 23;9(11):1596–600. doi: 10.1080/23802359.2024.2432367 (PMC11587729; doi:10.1080/23802359.2024.2432367)

Figure S1. The read coverage depth map of the assembled genome.

Figure S2. Schematic maps of cis-splicing genes in the chloroplast genome of *L. paradoxum*. Maps generated using CPGView. The gene names are shown on the left, and the gene structures are on the right.

Figure S3. Schematic maps of the trans-spliced gene *rps12*. Maps generated using CPGView.

Figure S1


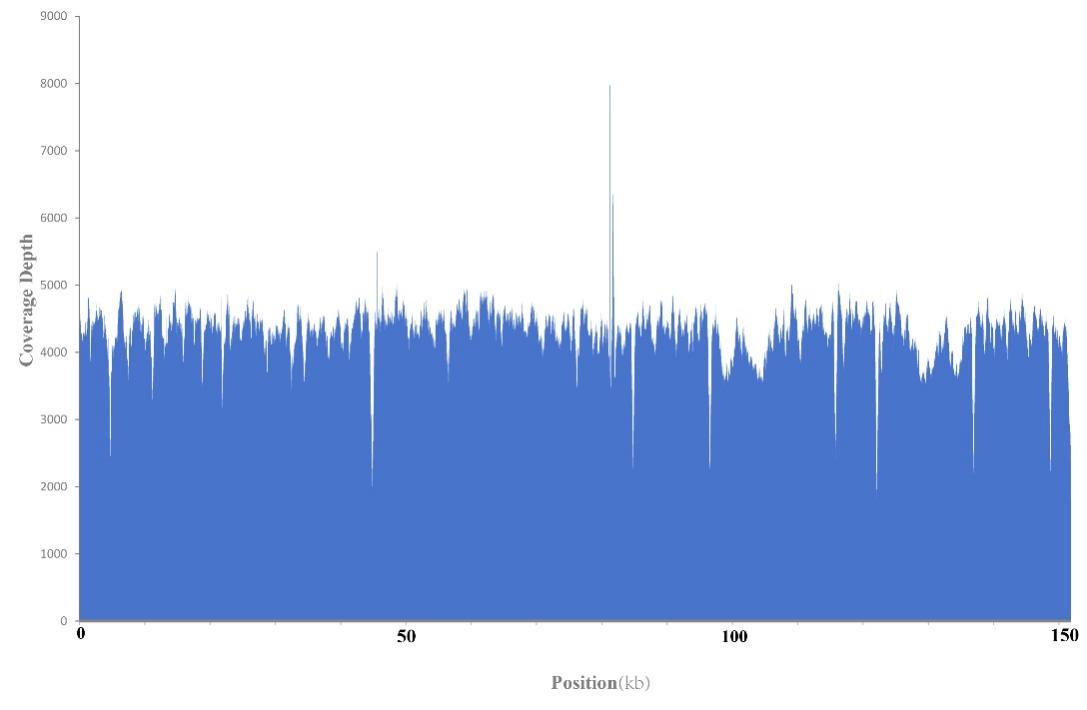


Figure S2


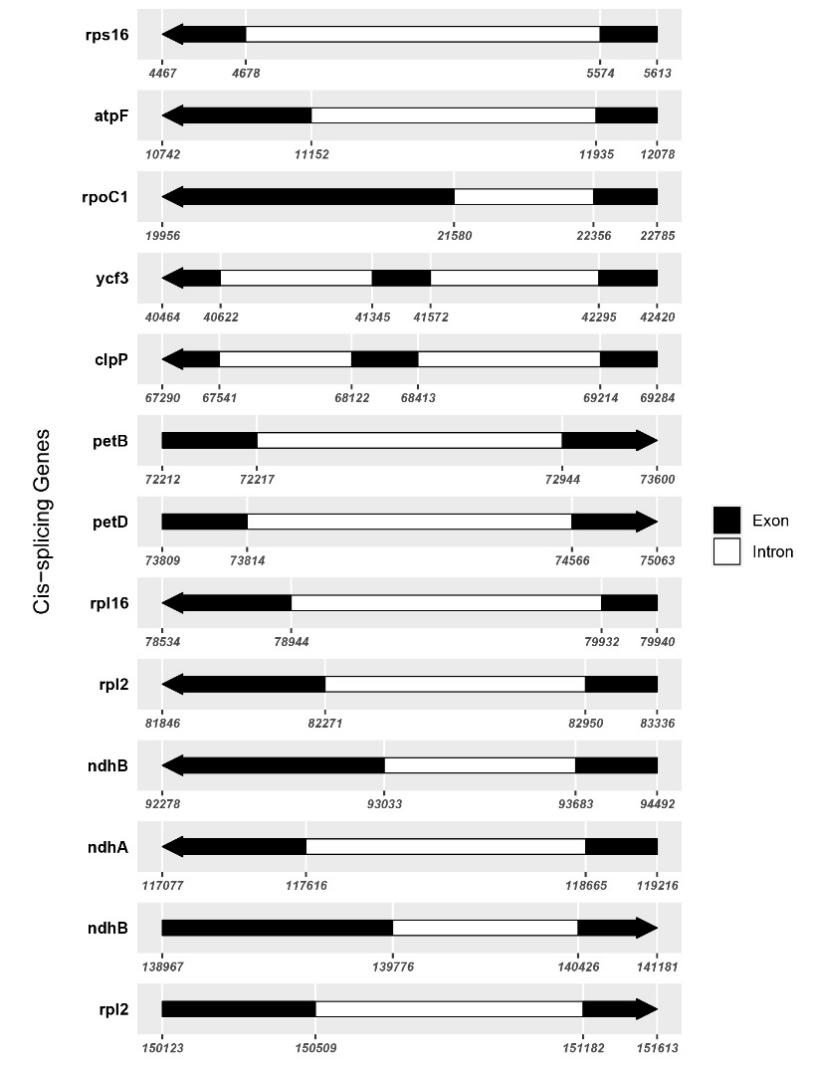


Figure S3


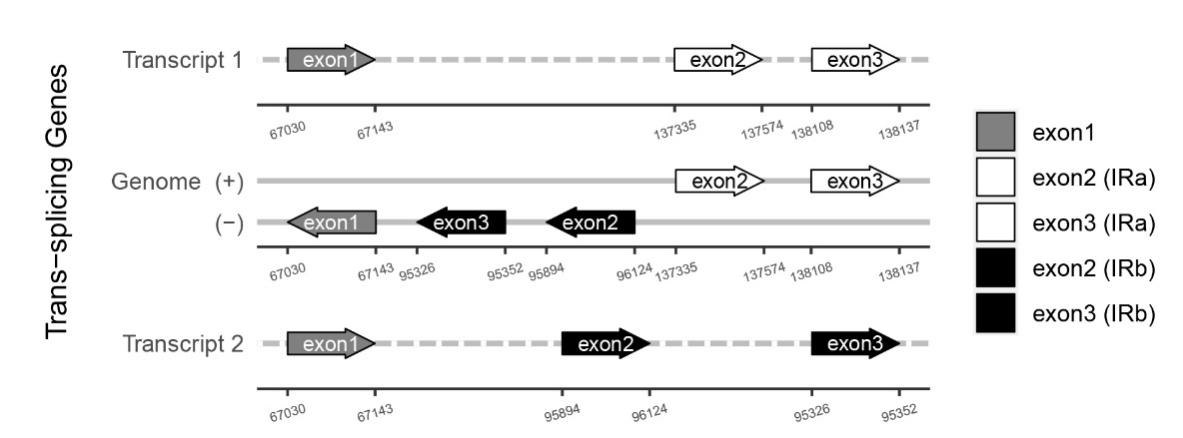

Supplement: Supplemental Material [file TMDN_A_2432367_SM5277.docx]
